# The Association between Impaired Glucose Regulation and Prognosis of Chinese Patients with Intracerebral Hemorrhage

**DOI:** 10.1038/srep36220

**Published:** 2016-10-31

**Authors:** Shichao Sun, Yuesong Pan, Xingquan Zhao, Liping Liu, Hao Li, Yan He, Li Guo, Yilong Wang, Yongjun Wang

**Affiliations:** 1 Department of Neurology, The Second Hospital, Hebei Medical University, Shi Jiazhuang, Hebei Province, China; 2 Department of Neurology, Beijing Tiantan Hospital, Capital Medical University, Beijing, China; 3 Department of Epidemiology and Health Statistics, School of Public Health, Capital Medical University, Beijing, China; 4 China National Clinical Research Center for Neurological Diseases, Beijing, China; 5 Center of Stroke, Beijing Institute for Brain Disorders, Beijing, China; 6 Beijing Key Laboratory of Translational Medicine for Cerebrovascular Disease, Beijing, China

## Abstract

This study aimed at observing the influence of impaired glucose regulation (IGR) on 1-year outcomes in patients with intracerebral hemorrhage (ICH). Patients hospitalized for ICH from 2008 to 2009 were recruited consecutively at 35 centres across China. A standard oral glucose tolerance test at day 14 ± 3 after stroke onset or before discharge was performed to identify IGR. The outcomes were death (modified Rankin scale [mRS] score of 6), dependency (mRS score of 2 to 5) and poor outcome (mRS score of 2 to 6) at 1 year. Cox proportion hazard model for death and logistic regression model for dependency and poor outcome were performed to investigate the influence of IGR on 1-year outcomes. A total of 288 non-diabetic ICH patients were included in this analysis, among which 150 (52.1%) were IGR. IGR was associated with 1-year dependency (adjusted odds ratio [OR] 2.18, 95% confidence interval [CI], 1.19–3.99; P = 0.01) and poor outcome (adjusted OR 2.17; 95% CI, 1.24–3.80; P = 0.007) of patients with ICH. However, IGR showed no significant association with 1-year death (adjusted hazard ratio 1.49, 95% CI, 0.60–3.67; P = 0.39). IGR was independently associated with 1-year poor outcome of ICH in Chinese patients, with more important influence on dependency than death.

Impaired glucose regulation (IGR), also known as pre–diabetes mellitus (pre-DM), is an intermediate metabolic state between normal glucose tolerance (NGT) and diabetes mellitus (DM), which comprises impaired fasting glucose (IFG) and/or impaired glucose tolerance (IGT)^1^,^2^. There is a growing recognition of IGR as a risk factor for developing stroke^3^. However, evidence on the relationship between IGR and stroke outcomes is still limited. Although previous studies have illustrated the deleterious effect of IGR on ischemic stroke outcomes^4^,^5^, information on IGR and intracerebral hemorrhage (ICH) outcomes was scarce^6^.

Our study aimed to investigate the association between IGR and 1-year outcomes of ICH patients in a representative cohort study named Abnormal gluCose Regulation in patients with acute strOke acroSS China (ACROSS-China).

## Results

### Characteristics at baseline

**Figure 1:**
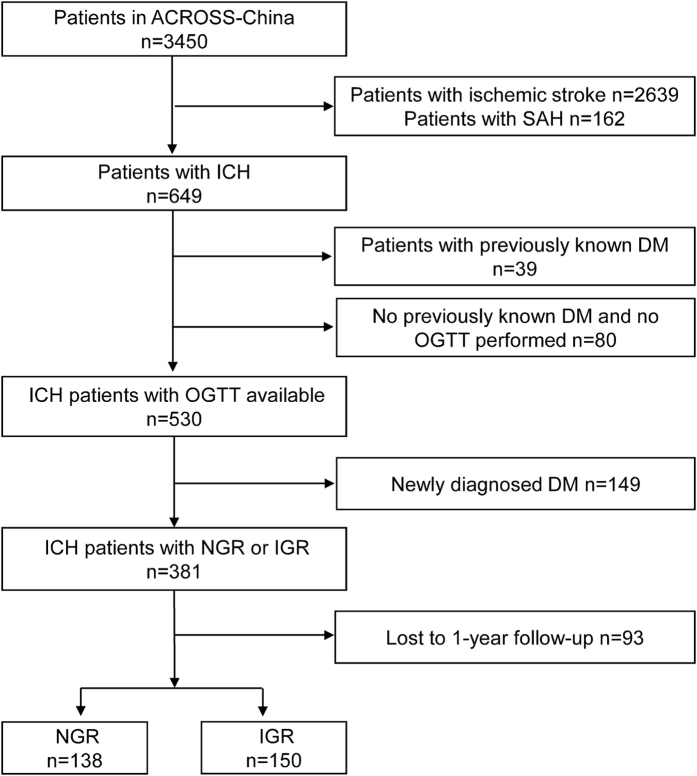
Patient flow. ACROSS-China indicates the study of Abnormal gluCose Regulation in patients with acute strOke acroSS China.; SAH, subarachnoid hemorrhage; ICH, intracerebral hemorrhage; DM, diabetes mellitus; OGTT, oral glucose tolerance test; NGR, normal glucose regulation; IGR, impaired glucose regulation.

The ACROSS-China study enrolled 649 ICH patients, among which, 39 patients had previously known DM and 530 patients without previously known diabetes were performed with oral glucose tolerance test (OGTT). The causes of the other 80 patients who had neither previously known diabetes nor available OGTT results included coma, alimentary tract hemorrhage, and dysphagia. Among the 530 patients, 189 cases (35.7%) were IGR and 149 cases (28.1%) were newly diagnosed DM (NDM). Patients with NDM were further excluded. A total of 93 patients lost to follow-up at 1 year and the remaining 288 patients were included in this analysis (Fig. 1). The clinical characteristics and proportion of IGR (41.9% versus 52.1%; P = 0.09) were well balanced between patients included and those lost to follow-up (Table 1).

The ages of the participants in the analysis ranged from 26 to 90 years (mean 58 ± 13 years) and 95 (33.1%) were female. IGR was present in 150 (52.1%) patients, among which 9 (6.0%) were isolated IFG, 125 (83.3%) were isolated IGT and 16(10.7%) were complex IGT (IFG and IGT). Comparing with those with NGR, patients with IGR had higher HbA1c and fasting plasma glucose (FPG) on admission (Table 2).

### Association between IGR and outcomes of ICH

Univariable analyses of risk factors for 1-year outcomes of ICH are presented in Table 3. Outcomes of ICH were associated with body mass index, hematoma location, National Institutes of Health Stroke Scale (NIHSS) score on admission and Glasgow Coma Scale (GCS) score on admission. The 1-year outcomes of patients with IGR or NGR were compared and presented in Table 4. Patients with IGR had higher proportion of dependency (43.9% versus 25.6%, P = 0.002) and poor outcome (50.0% versus 29.9%, P < 0.001), comparing with patients with NGR. After adjusting for potential covariates, IGR was associated with 1-year dependency (adjusted odds ratio [OR] 2.18; 95% confidence interval [CI], 1.19–3.99; P = 0.01) and poor outcome (adjusted OR 2.17; 95% CI, 1.24–3.80; P = 0.007). However, there was no significant association between IGR and death (10.7% versus 5.8%; adjusted Hazard ratio [HR] 1.49; 95% CI, 0.60–3.67; P = 0.39).

## Discussion

Our study indicated that IGR after ICH was associated with 1-year poor outcome and dependency of Chinese patients with ICH. No clear association was observed between IGR and 1-year mortality.

Most studies about IGR focused on the prevalence of IGR in patients with stroke^7^,^8^ and the prognosis value of IGR in outcomes of ischemic stroke^4^,^9^. For ICH patients, there was only 1 previous study on IGR and outcomes in a mixed stroke population including 168 patients with ICH^6^, showing the adverse influence of IGR on short-term unfavourable outcome. However, the definition of IGR was based on fasting glucose levels measured on day 2–4 after admission in that study. In our study, a standard OGTT was performed on day 14 ± 3 after onset or before discharge, and patients in acute stage of ICH were not performed with the test. Therefore, the influence of stress response on IGR was relatively minor, which emphasized the direct effect of abnormal glucose regulation on outcomes.

Isolated IGT accounted for a large proportion of IGR in our study, which was consistent with the characteristic that isolated IGT was common among Chinese patients with IGR^10^. Considering the distinct pathophysiological mechanisms between IGT and IFG^11^, the prognosis utility of IGT or IFG might be different. Unfortunately, our study failed to invest this point due to limited sample size.

The association of IGR and poor outcome in ICH could be explained by several pathophysiological disturbances. First, insulin resistance, as a major feature of IGR, is a state of decreased responsiveness to normal circulating levels of insulin, which could block the insulin signaling in neurons^12^. Previous studies have documented the increased neuronal damage with blocked insulin signaling^13^,^14^. Second, insulin resistance may frequently be accompanied by hyperinsulinemia. Exposure to hyperinsulinemia rather than hyperglycemia, has been proven to decrease the responsiveness of Akt activation^15^. Akt is a serine/threonine protein kinase involving in many signaling pathways to regulate cellular nutrient metabolism, cell growth, apoptosis and survival, the decreased activation of which may increase neuronal damage and thereby adversely influence functional recovery^16^,^17^,^18^,^19^. This may explain why IGR could influence functional outcome though the glucose was not remarkably increased. Third, insulin-like growth factor I (IGF-I), an important endogenous neurotrophic factors expressed in adult brain, was proved to reduce cell loss and improve long-term neurological function in animal models^20^,^21^. Previous study has reported that the stroke outcome could be impacted by IGF-I serum levels^22^. IGF-I could be suppressed by hyperinsulinemia and hence to influence the recovery and regenerative of damaged brain tissue^23^.

Our study shows inconsistent influence of IGR after ICH on dependency and death. The possible explanations are listed as follows. First, the majority of death after ICH occurs in the first month after ICH^24^,^25^,^26^. The harmful influence of IGR on brain, such as increasing neuronal damage and inhibiting neuronal repair and regeneration, may not be able to induce a statistically mortality differences in such a short time. Second, most early death after ICH results from the direct neurologic consequences of hemorrhage. It may be unable to statistically evaluate the relatively “minor” influence of IGR on death because its potential contributors to death is to some extent obscured by the hemorrhage severity-associated predictive factors such as the hematoma volume, location and neurologic function level^27^. However, for those survivors, it may be possible to distinguish the independent contribution of “minor” prognostic factors and hence the association between IGR and dependency was observed.

In our study, no clear association was observed between IGR and 1-year mortality. However, previous result from ACROSS-China indicated that IGR predicted 1-year mortality of Chinese patients with ischemic stroke. The possible explanation for the contrary death prognosis between ischemic and hemorrhagic stroke is the timing of death. As we mentioned above, it may take time for IGR to show its contribution to death. Comparing with ischemic stroke, the majority of death after ICH occurs in the earlier stage^24^,^25^,^26^,^28^ and mainly depends on factors such as the hematoma volume and location^27^. Under the obscuration of early death associated factors and short duration, it may be difficult for IGR to present a significant contribution to death in ICH. While for ischemic stroke, with relatively low early mortality, the contribution of IGR to death may be able to present statistically in a relatively long time.

The prevalence of IGR was high (26.3–32.8%) in patients with ICH^29^,^30^. Therefore, the association between IGR and clinical outcomes of ICH patients deserved investigation. To our knowledge, this is the first study reported the adverse influence of IGR on long-term clinical outcomes in solely ICH patients. The glucose management for patients with ICH in the current guidelines focused on the control of hyperglycemia, which didn’t mention the detection and intervention of IGR^31^. Considering its adverse effect on poor outcome, IGR deserves clinical concern in patients with ICH. Previous trial evaluating IGR intervention in non-diabetic patients with ischemic stroke and insulin resistance has showed a benefit effect on stroke recurrence^32^. It is likely, though unproven, that medical intervention, which were typically used in patients with established diabetes, may improve the functional outcome of patients with ICH and IGR. We recommend the routine screening of IGR with standard OGTT in ICH patients and hope to get more information about the effectiveness of IGR intervention on ICH outcomes in future randomized controlled trials.

Several limitations must be acknowledged. First, though it was a nationwide cohort, the study sample of ICH was relatively small and confined to Chinese population. The applicability of our conclusion merits further confirmation. Second, information on 1-year follow-up was available in 75.6% of patients with NGR or IGR in this study. However, baseline characteristics of patients with and without follow-up information were well-balanced. Third, it was unclear that IGR was ICH-induced or resulted from undiagnosed abnormal glucose regulation before ICH. However, the significant higher HbA1c level in IGR group than NGR group might indicate the abnormal glucose regulation existed before ICH onset. Finally, it was uncertain that IGR after ICH was persistent or transient. Whether the influence of IGR on outcomes could be altered by transformation of glucose metabolism status needed to be clarified in subsequent studies.

In conclusion, our study indicates that IGR was independently associated with 1-year poor outcome of ICH in Chinese patients, with more important influence on dependency than death.

## Materials and Methods

### Study Population

The study population was derived from the study of ACROSS-China, the baseline characteristics and recruiting criteria of which have been described in detail elsewhere^29^. In brief, ACROSS-China was a nationwide, multicenter and prospective cohort study, aimed at investigating the prevalence and influence of abnormal glucose regulation among patients hospitalized with acute stroke. Patients with ischemic stroke, ICH, or subarachnoid hemorrhage (SAH) within 14 days after onset were recruited consecutively from 35 participating hospitals across China from August 2008 to October 2009. A standard OGTT was performed in all the participants without previously known DM at the day 14 ± 3 after stroke onset or before discharge according to the World Health Organization (WHO) criteria^33^. ACROSS-China was performed in accordance with the guidelines of the Helsinki Declaration. The protocol and data collection were approved by the Ethics Committees at Beijing Tiantan Hospital and all participating hospitals. Written informed consent was obtained from each participant or his/her designated relatives.

Acute stroke was diagnosed according to WHO criteria^34^ combined with brain CT or MRI confirmation. For the current study, patients with ischemic stroke and SAH were excluded. We also excluded patients with previously known DM, no OGTT performed, NDM based on OGTT results and patients lost to follow-up.

### Data Collection and Variable Definition

Demographic characteristics, clinical information, status on admission including stroke severity and blood pressure values, hematoma locations, laboratory values on admission, treatment during hospitalization and OGTT results were collected from the database. Stroke severity was measured using the initial NIHSS score and GCS score.

The definition of NGR, IGR and NDM were based on the OGTT results. According to the WHO criteria^35^, IGR was defined as having isolated IFG (FPG≥6.1 mmol/L and <7.0 mmol/L, meanwhile, 2-h plasma glucose [2-h PG] <7.8 mmol/L), or isolated IGT (FPG <6.1 mmol/L, meanwhile, 2-h PG ≥7.8 mmol/L and <11.1 mmol/L), or complex IGT (FPG ≥6.1 mmol/L and <7.0 mmol/L, meanwhile, 2-h PG ≥7.8 mmol/L and <11.1 mmol/L). NGR was defined as FPG <6.1 mmol/L and 2-h PG <7.8 mmol/L. NDM was defined as FPG ≥7.0 mmol/L and/or 2-h PG ≥11.1 mmol/L. Patients with self-reported physician diagnosis of DM or hypoglycemic treatment before ICH were classified as those with previously known DM.

### Outcome Measures

The outcomes were all-cause death (modified Rankin scale [mRS] score of 6), dependency (mRS score of 2 to 5) and poor outcome defined as mRS score of 2 to 6 at 1 year^36^. The 1-year follow-up of participants were conducted by trained research personnel at Beijing Tiantan Hospital. The mRS score at 1 year after onset of ICH were recorded through telephone interview. A death certificate from the local citizen registry or the attended hospital was used to confirm the case fatality.

### Statistical Analysis

Baseline demographic and clinical characteristics were expressed as mean (standard deviation) or median (interquartile range) for continuous variables and as percentages for categorical variables. The chi-square test for categorical variables and Mann–Whitney test for continuous variables were used as needed. Cox proportion hazard model for death and logistic regression model for dependency and poor outcome were performed both in univariable and multivariable analyses to show the association between IGR and outcomes. HRs and ORs with 95%CI were calculated. Age, gender and all significant baseline variables in the univariable analysis were included in the multivariable analysis. A 2-sided P value < 0.05 was set as the level for statistical significance. All analyses were performed with SAS software version 9.4 (SAS Institute Inc, Cary, NC, USA).

## Additional Information

**How to cite this article**: Sun, S. *et al*. The Association between Impaired Glucose Regulation and Prognosis of Chinese Patients with Intracerebral Hemorrhage. *Sci. Rep*. **6**, 36220; doi: 10.1038/srep36220 (2016).

**Publisher’s note**: Springer Nature remains neutral with regard to jurisdictional claims in published maps and institutional affiliations.

## Figures and Tables

**Table 1:** Baseline characteristics of patients included in this study versus those lost to follow up.

Variables	Included (n = 288)	Lost to follow up (n = 93)	P Value
Age (year), mean (SD)	57.5 (13.2)	57.0 (13.2)	0.74
Male, n (%)	192 (66.9)	60 (64.5)	0.67
BMI (kg/m^2^), mean (SD)	24.6 (4.2)	23.8 (2.8)	0.19
Medical history, n (%)			
Hypertension	188 (65.3)	57 (61.3)	0.49
Dyslipidemia	10 (3.5)	3 (3.2)	1.00
Coronary heart disease	23 (8.0)	6 (6.5)	0.63
Atrial fibrillation	6 (2.1)	1 (1.1)	0.85
Stroke	28 (10.6)	4 (4.8)	0.11
Pre-disability	2 (0.7)	4 (4.3)	0.051
On admission status			
NIHSS score, median (IQR)	6 (2–12)	8 (4–11)	0.20
GCS score, median (IQR)	15 (13–15)	15 (13–15)	0.96
SBP (mmHg), mean (SD)	156 (24)	153 (22)	0.46
DBP (mmHg), mean (SD)	92 (15)	91 (14)	0.73
Hematoma location, n (%)			0.18
Lobes	41 (14.2)	5 (5.4)	
Basal ganglia region	203 (70.5)	72 (77.4)	
Brain stem	15 (5.2)	8 (8.6)	
Cerebellum	17 (5.9)	3 (3.2)	
Intraventricular extension	7 (2.4)	3 (3.2)	
TG on admission (mmol/L), median (IQR)	1.4 (1.1–2.0)	1.4 (1.0–1.9)	0.24
HbA1c on admission (%),median (IQR)	5.5 (5.0–6.0)	5.4 (4.8–5.7)	0.11
FPG on admission (mmol/L), median (IQR)	5.4 (4.8–6.1)	5.5 (4.7–6.3)	0.77
FPG at 14d (mmol/L), median (IQR)	4.9 (4.5–5.5)	5.0 (4.5–5.5)	0.07
2-h PG at 14d (mmol/L), median (IQR)	7.7 (6.7–9.2)	7.2 (6.3–8.8)	0.05

SD indicates standard deviation; IQR, interquartile range; BMI, body mass index; NIHSS, National Institute of Health Stroke Scale; GCS, Glasgow Coma Scale; SBP, systolic blood pressure; DBP, diastolic blood pressure; TG, triglyceride; and HbA1c, glycosylated hemoglobin.

**Table 2:** Baseline characteristics of patients with NGR versus IGR.

Variables	Total (n = 288)	NGR (n = 138)	IGR (n = 150)	P Value
Age (year), mean (SD)	57.5 (13.2)	56.2 (14.1)	58.7 (12.2)	0.12
Male, n (%)	192 (66.9)	98 (71.5)	94 (62.7)	0.11
BMI (kg/m^2^), mean (SD)	24.6 (4.2)	24.5 (5.0)	24.6 (3.4)	0.50
Medical history, n (%)				
Hypertension	188 (65.3)	90 (65.2)	98 (65.3)	0.98
Dyslipidemia	10 (3.5)	2 (1.5)	8 (5.3)	0.14
Coronary heart disease	23 (8.0)	16 (11.6)	7 (4.7)	0.03
Atrial fibrillation	6 (2.1)	2 (1.5)	4 (2.7)	0.76
Stroke	28 (10.6)	17 (13.0)	11 (8.3)	0.21
Pre-disability	2 (0.01)	2 (0.01)	0 (0.0)	0.23
On admission status				
NIHSS score, median (IQR)	6 (2–12)	6 (2–11)	7 (2–13)	0.09
GCS score, median (IQR)	15 (13–15)	15 (13–15)	15 (12–15)	0.35
SBP (mmHg), mean (SD)	156 (24)	156 (26)	155 (22)	0.82
DBP (mmHg), mean (SD)	92 (15)	92 (15)	92 (14)	0.92
Hematoma location, n (%)				0.12
Lobes	41 (14.2)	16 (11.6)	25 (16.7)	
Basal ganglia region	203 (70.5)	93 (67.4)	110 (73.3)	
Brain stem	15 (5.2)	9 (6.5)	6 (4.0)	
Cerebellum	17 (5.9)	11 (8.0)	6 (4.0)	
Intraventricular extension	7 (2.4)	6 (4.4)	1 (0.7)	
TG on admission (mmol/L), median (IQR)	1.4 (1.1–2.0)	1.3 (0.9–1.9)	1.5 (1.2–2.0)	0.05
HbA1c on admission (%),median (IQR)	5.5 (5.0–6.0)	5.3 (4.7–5.8)	5.7 (5.2–6.2)	<0.001
FPG on admission (mmol/L), median (IQR)	5.4(4.8–6.1)	5.1(4.6–5.8)	5.6(5.1–6.4)	<0.001
FPG at 14d (mmol/L), median (IQR)	4.9 (4.5–5.5)	4.7 (4.3–5.1)	5.1 (4.7–5.7)	<0.001
2-h PG at 14d (mmol/L), median (IQR)	7.7 (6.7–9.2)	6.7 (5.8–7.2)	9.1 (8.4–10.1)	<0.001
Medication in hospital, n (%)				
Oral hypoglycemic agents	2 (0.7)	0 (0.0)	2 (1.3)	0.52
Insulin administration	10 (3.5)	5 (3.6)	5 (3.3)	1.0
Antihypertensive therapy	193 (67.0)	95 (68.8)	98 (65.3)	0.53
In-hospital pulmonary infection, n (%)	40(13.9)	16(11.6)	24(16.0)	0.28
In-hospital urinary infection, n (%)	10 (3.5)	3 (2.2)	7 (4.7)	0.41

P values refer to the comparisons between IGR and NGR groups. NGR indicates normal glucose tolerance; IGR, impaired glucose regulation; SD, standard deviation; IQR, interquartile range; BMI, body mass index; NIHSS, National Institute of Health Stroke Scale; GCS, Glasgow Coma Scale; SBP, systolic blood pressure; DBP, diastolic blood pressure; TG, triglyceride; HbA1c, glycosylated hemoglobin; FPG, fasting plasma glucose; and 2-h PG, 2-hour plasma glucose.

**Table 3:** Baseline characteristics of patients according to 1-year outcomes.

Variables	No (n = 264)	Death	P Value	No (n = 169)	Dependency	P Value	No (n = 169)	Poor outcome	P Value
		Yes (n = 24)			Yes (n = 90)			Yes (n = 114)	
Age (year), mean (SD)	57.1 (13.1)	62.7 (13.1)	0.048	55.8 (13.0)	58.6 (12.9)	0.11	55.8 (13.0)	59.4 (13.0)	0.03
Male, n (%)	176 (66.9)	16 (66.7)	0.98	119 (70.8)	52 (57.8)	0.03	119 (70.8)	68 (59.7)	0.05
BMI (kg/m^2^), mean (SD)	24.7 (4.3)	22.9 (2.7)	0.02	24.8 (4.4)	24.7 (4.1)	0.75	24.8 (4.4)	24.3 (3.9)	0.22
Medical history, n (%)									
Hypertension	174 (65.9)	14 (58.3)	0.46	111 (65.7)	60 (66.7)	0.87	111 (65.7)	74 (64.9)	0.89
Dyslipidemia	9 (3.4)	1 (4.2)	0.59	7 (4.1)	2 (2.2)	0.65	7 (4.1)	3 (2.6)	0.73
Coronary heart disease	19 (7.2)	4 (16.7)	0.21	12 (7.1)	6 (6.7)	0.90	12 (7.1)	10 (8.8)	0.61
Atrial fibrillation	4 (1.5)	2 (8.3)	0.08	1 (0.6)	2 (2.2)	0.58	1 (0.6)	4 (3.5)	0.17
Stroke	26 (10.8)	2 (8.7)	1.0	17 (11.1)	9 (10.7)	0.93	17 (11.1)	11 (10.3)	0.83
Pre-disability	2 (0.8)	0 (0.0)	1.0	1 (0.6)	1 (1.1)	1.0	1 (0.6)	1 (0.9)	1.0
Hematoma location, n (%)			0.73			0.02			0.07
Lobes	37 (14.0)	4 (16.7)		27 (16.0)	8 (8.9)		27 (16.0)	12 (10.5)	
Basal ganglia region	186 (70.5)	17 (70.8)		110 (65.1)	73 (81.1)		110 (65.1)	90 (79.0)	
Brain stem	15 (5.7)	0 (0.0)		8 (4.7)	7 (7.8)		8 (4.7)	7 (6.1)	
Cerebellum	16 (6.1)	1 (4.2)		14 (8.3)	2 (2.2)		14 (8.3)	3 (2.6)	
Intraventricular extension	6 (2.3)	1 (4.2)		6 (3.6)	0 (0.0)		6 (3.6)	1 (0.9)	
On admission status									
NIHSS score, median (IQR)	6 (2–11)	13 (6–19)	0.002	3 (1–7)	11 (7–15)	<0.001	3 (1–7)	11 (6–15)	<0.001
GCS score, median (IQR)	15 (13–15)	14 (10–15)	0.06	15 (14–15)	13 (11–15)	<0.001	15 (14–15)	14 (11–15)	<0.001
SBP (mmHg), mean (SD)	156 (24)	156 (23)	0.73	156 (25)	155 (22)	0.90	156 (25)	155 (22)	0.97
DBP (mmHg), mean (SD)	92 (15)	91 (13)	0.76	92 (16)	92 (14)	0.92	92 (16)	92 (14)	0.84

SD indicates standard deviation; IQR, interquartile range; BMI, body mass index; NIHSS, National Institute of Health Stroke Scale; GCS, Glasgow Coma Scale; SBP, systolic blood pressure and DBP, diastolic blood pressure.

**Table 4:** Association between IGR and 1-year outcomes.

Outcomes	Events, n (%)		Unadjusted		Adjusted	
	NGR	IGR	OR/HR(95% CI)*	P Value	OR/HR(95%CI)*	P Value
Death	8 (5.8)	16 (10.7)	1.87 (0.80–4.38)	0.15	1.49 (0.60–3.67)†	0.39
Dependency	33 (25.6)	57 (43.9)	2.27 (1.34–3.84)	0.002	2.18 (1.19–3.99)‡	0.01
Poor outcome	41 (29.9)	73 (50.0)	2.34 (1.44–3.82)	<0.001	2.17 (1.24–3.80)§	0.007

^*^Patients with NGR was referent group. OR for the outcome of dependency and poor outcome and HR for the outcome of death.

^†^Adjusted for gender, age, body mass index, and National Institute of Health Stroke Scale score on admission.

^‡^Adjusted for gender, age, hematoma location, National Institute of Health Stroke Scale score on admission, and Glasgow Coma Scale score on admission.

^§^Adjusted for gender, age, National Institute of Health Stroke Scale score on admission, and Glasgow Coma Scale score on admission.

NGR indicates normal glucose regulation; IGR, impaired glucose regulation; OR, odds ratio; HR, hazard ratio and CI, confidence interval.
